# Tracheal and cloacal bacterial diversity of red listed Eastern Imperial Eagle (*Aquila heliaca*)

**DOI:** 10.3389/fmicb.2025.1477032

**Published:** 2025-05-09

**Authors:** Marine Murtskhvaladze, Levan Ninua, Nika Budagashvili, Ekaterine Tevdoradze, Zurab Gurgenidze, Adam Kotorashvili, Nato Kotaria, Alexander Gavashelishvili, Zurab Javakhishvili

**Affiliations:** ^1^Faculty of Natural Sciences and Medicine, Ilia State University, Tbilisi, Georgia; ^2^R. L. Lugar Center, L. Sakvarelidze National Center for Disease Control and Public Health, Tbilisi, Georgia; ^3^SABUKO (Society for Nature Conservation), Tbilisi, Georgia

**Keywords:** Eastern Imperial Eagle, tracheal bacterias, cloacal bacterias, 16S rRNA, conservation

## Abstract

This study aimed to improve knowledge of raptor microbiomes by providing the first description of tracheal and cloacal bacterial diversity of Eastern Imperial Eagles (*Aquila heliaca*). To date, only few studies are available and they are carried out mainly on captive birds. The Eastern Imperial Eagle is species of significant conservation concern and, therefore, characterization microbiota contributes valuable information to the field of avian microbiology and aids in conservation efforts for this threatened species, moreover, identification of avian and human pathogens within microbial communities and evaluation of potential threats to birds, humans, and other species are crucial for sustainably balancing the wellbeing of ecosystems, 3,500 OTUs were identified from each sample supported by ∼2.8 Million sequence reads. The tracheal and cloacal microbiomes were dominated by *Gammaproteobacteria* (67.5%), *Bacilli* (43.8%), and *Negativicutes* (22.0%). We detected dissimilarities between cloacal (unique 440 OTUs) and tracheal (337 unique OTUs) samples, and significant evidence of moderate positive monotonic relationship between cloacal and tracheal bacterial communities. No significant differences between individuals from different nests. *Aquila heliaca* can serve as an indicator of presence of bacterial species in its respective habitats. Efforts aiming at protection of red-listed birds may not presently prioritize microbiome considerations but integrating microbiome research into conservation strategies could yield significant benefits.

## Introduction

The Eastern Imperial Eagle (*Aquila heliaca*) is a threatened species in Georgia. Up to 20 pairs breed in the east of the country (Galvez et al., 2005; Javakhishvili et al., 2020; Budagashvili, 2024). The species is listed as vulnerable (VU) (IUCN, 2016) and is declining over the entire breeding range (BirdLife, 2023). The main causes of mortality are poaching, collisions with power lines, habitat degradation, and the reduction of the habitual prey (Rudnick et al., 2008; Bragin et al., 2021; Budagashvili, 2024). Despite concerted legislative efforts, the regressive trend persists.

While biological and ecological studies have shed light on various aspects of *A. heliaca*’s life history (Hák et al., 2007; Katzner et al., 2014; Bekmansurov et al., 2015; Bragin et al., 2021), the microbial landscape within this species remains unexplored. The prevalence and incidence of infectious diseases in these birds remain poorly understood. Characterization and monitoring of microbiomes is emerging as a tool in conservation, particularly for the management of endangered species (Bahrndorff et al., 2016; West et al., 2019; Dallas and Warne, 2023). This approach provides insights into the relationships between the microbial communities inhabiting various species and their environment, offering valuable data that can help guide conservation strategies (Song et al., 2020; Dallas and Warne, 2023). As a result, the role of microbiomes in conservation biodiversity and supporting species at risk of extinction is being increasingly recognized. Undertaking relevant surveillance for diseases that may pose a threat to populations of birds of prey is a key aspect of preparing national or regional raptor conservation and management strategies (Kovács and Williams, 2012). Monitoring diseases and assessing their role in the effective conservation of *Aquila heliaca* is widely acknowledged by scientists working on the conservation of the species (Heredia, 1996; Horváth et al., 2006; Budagashvili, 2024). Although studies on microbiome of wildlife are increasing, most of these studies have focused on mammals (Colston and Jackson, 2016; Moeller and Sanders, 2020; Jeong et al., 2021), whereas bird species remain an exception (Araghi et al., 2015; Grond et al., 2018; Capunitan et al., 2020 Zhao et al., 2022).

The interactions between different taxa within a microbiome are complex and multidirectional, and actively influence the overall health and wellbeing of the host species. Microbial composition is host specific and undergoes dynamic change over host’s lifetime, shaped by various factors: environmental conditions, dietary patterns and physiological state (Sekirov et al., 2010). The microbial community inhabiting in an organism plays a fundamental role in key ecological processes that are essential for resilience and survival. Studies indicate that it is associated with disease resistance, also influences host lifespan, reproductive success, and adaptive capacities to changes in the environment (Casadevall and Pirofski, 2000; Muegge et al., 2011; Stenkat et al., 2014). Mycotic, bacterial and viral species are beneficial, while some contribute to essential physiological processes and host adaptations, others cause infectious diseases and are identified as a threat of biodiversity (Daszak et al., 2000; Brilhante et al., 2012; West et al., 2019; Cunningham et al., 2017). The interconnection between microbial ecology and host health, necessitates a holistic approach to the conservation of threatened species (Bordenstein and Theis, 2015; Bahrndorff et al., 2016). Disease outbreaks can lead to disruption of ecological interactions, affect reproductive capabilities, alter predator-prey dynamics by affecting the health and behavior of species, decline population sizes, and even drive species to extinction (Smith et al., 2006; Hohenlohe et al., 2021). For endangered or vulnerable species, these microbial interactions are especially significant, as they can directly affect their ability to cope with environmental stressors and diseases. This underscores the importance of studying microbial communities within threatened species, as a lack of knowledge can have far-reaching consequences, thus, a deeper understanding of microbiome dynamics offers a promising avenue for enhancing conservation efforts by supporting the health, resilience, and long-term survival of the species.

Besides well-assessed various molecular methods (target sequencing, whole genome sequencing), to date we have a poor understanding of the evolutionary and ecological processes relevant to microbiome composition and function, especially in wild organisms and non-model taxa. Compounding these challenges is the difficulty in collecting biological samples, as rare niches in remote geographical locations present obstacles to efficient sampling. Microbiome studies of threatened species are of increasing concern for development management strategies and survival success of the species (Bahrndorff et al., 2016; West et al., 2019; Dallas and Warne, 2023). Incorporating such studies into conservation strategies can enhance our understanding of the complex interplay between organisms and their microbial communities, ultimately contributing to the successful management and survival.

Birds of prey cover hundreds of kilometers every year during migration and tend to spread emerging infectious diseases (Movalli et al., 2018). Raptors, due to their role as both predators and scavengers, can become reservoirs for a range of potentially zoonotic agents (Vidal et al., 2017; Rossi et al., 2021; Vogt, 2022). Obligatory migrant Eastern Imperial Eagle might be carriers of microbial pathogens and be potential spreaders to other species, even in humans e.g., droppings containing strains of *Clostridium* and *Campylobacter* spp. (Brilhante et al., 2012; Jurado-Tarifa et al., 2016). Studies of bacterial infections and carriage rates in raptors are limited and are focused on disease outbreaks in domestic birds and pet birds due to the economic value of the industry. The lack of interest and low commercial value have resulted in a limited understanding of the role of *Aquila* species as reservoirs and zoonotic disease vectors. Understanding the potential for the spread of avian bacterial pathogens from migrant raptors could provide a valuable model for estimating the risks associated with disease transmission among wild birds and other taxa. Focus on spreading bacteria in poultry has restrictions, as findings from domestic birds may not capture the full spectrum of transmission routes and other types of avian pathogens might not be screened.

The 16S rRNA sequencing method is a marker gene approach and comprehensively identifies all culturable and non-culturable as well as known and unknown microorganisms present in the upper respiratory tract and in the digestive system and hence, provides a better taxonomic resolution and genomic information on the microbial communities of host organisms (Dick et al., 2009; Johnson et al., 2019).

In Georgia, between 2016 and 2021, 18 chicks of Eastern Imperial Eagle were captured and tagged with GPS telemetry devices, 14 of these chicks died within the first year after hatching (cause of death undetermined) (Budagashvili, 2024). The primary objective of the study was to investigate the microbiome in nestling chicks in order to screen for pathogenic bacteria, which could potentially explain the high mortality rate among juvenile eagles. Given the inherent difficulty of catching and sampling wild (free-living) raptors, to minimize potential disturbances and the stress associated with handling, we analyzed tracheal and cloacal swabs from chicks. Although fecal samples are good sources for intestinal microbiome studies (Gao et al., 2021), the arid habitat of *A. heliaca* resulted in limited fecal material in nests, making chick sampling difficult and stressful.

Understanding the microbiome of juvenile Eastern Imperial Eagles is crucial, as it can offer insights into the health of these birds, potential pathogenic bacteria they may harbor, and how their microbiomes might influence their overall development and survival. In the first weeks of life, chicks of the Eastern Imperial Eagle primarily rely on regurgitated food provided by their parents. This feeding behavior not only ensures that the chicks’ nutritional needs are met, but also plays a crucial role in the establishment of their initial microbiome through the inoculation of microbiota from parents to offspring (Wang et al., 2018; Dietz et al., 2019; Bodawatta et al., 2022a).

By analyzing the bacterial composition of nestling chicks, we can gain valuable insights—though not an exact match—into the microbial communities, especially pathogenic organisms, present in their parents. This approach is particularly useful because studying adult, free-living raptors in their natural habitat presents significant challenges. As a result, examining the microbiota of the chicks provides an indirect, yet informative, glimpse into the microbial composition of the parent birds. By characterizing the microbiomes of the tracheal and cloacal environments in *A. heliaca*, we can identify avian and human prospective pathogens, assess the occurrence of microbial diseases and evaluate potential threats to the species, to humans and other birds. Considering how ecological factors, nesting sites, microclimate and age, might influence microbial communities, we can estimate indicators of the overall health of the eagles and provide recommendations for targeted conservation strategies for the population in Georgia.

## Materials and methods

### Sample collection

Six *A. heliaca* nests along the Iori River in a semi-arid habitat were sampled, representing 30% of the known Georgian population. Distances between nests ranged from 3.7 to 96.2 km. Seven chicks, approximately 8 weeks old and showing no visible health complications, were sampled from these nests. Sterile swabs were used to collect tracheal and cloacal samples, which were placed separately in sterile phosphate-buffered saline (PBS, pH 7.0). All samples were frozen before laboratory processing. No birds were killed or injured during sampling.

### Nucleic acid extraction and 16S rRNA amplification

Nucleic acids were extracted from tracheal and cloacal swabs using a MagMAX total nucleic acid isolation kit (Thermo Fisher Scientific) according to the manufacturer’s instructions, with a final 60 μL of elution volume. We determined the sex of each chick by amplifying the CHD1 gene fragment according to Fridolfsson and Ellegren (1999). Samples obtained from the trachea and cloaca were amplified and sequenced separately. The complete 1,500 bp 16S rRNA gene was amplified with primer pairs: CCTAYGGGNNGCNGCAG and GACTACNVGGGTMTCTAATCC (Patel, 2001). PCR was carried out with final concentrations of 1 μM of each primer, 0.3 mM dNTPs, 1.5 mM Mg2 +, 1 unit of Q5 High-Fidelity DNA Polymerase (Promega Inc.) and 1X buffer. The cycling conditions were as follows: initial denaturation at 95°C for 3 min followed by 30 cycles at 95°C for 40 s, 55°C 40 s, 72°C for 2 min. The amplicons were verified via 1% agarose gel electrophoresis, with SybrSafe staining. Each PCR reaction was repeated three times. The amplicons were mixed to ensure amplification of a substantial proportion of all possible microbial taxa.

16S rRNA amplicons were fragmented via enzymatic digestion using an NEBNext Ultra II FS kit according to the manufacturer’s instructions. The fragmented DNA was sized 350-650 bp segments using AMPure XP beads. DNA libraries were repaired, and Illumina-specific adapter sequences were ligated to each fragment. The samples were dual indexed and a second size selection step was performed. Purified DNA libraries were quantified using Tape Station DS1000 high sensitivity reagents (Agilent Technologies) and the samples were diluted to a concentration of 2 nM concentration. For the internal control, 1% PHiX DNA was used. The 12 pM denatured libraries were sequenced with a MiSeq Reagent Kit v3 (600-cycle) kit according to the manufacturer’s protocol.

### Data analysis

The Illumina base calling pipeline v1.40 was used for fluorescence image analysis and FasTQ formatted sequence data were obtained. Read-based taxonomic classification was performed with the EDGE web based graphic interface (Li et al., 2017) and Geneious prime.^1^ The sequences were quality controlled using FastQC v.0.12.1 (Andrews, 2010). Low quality reads, adapters, indexes and primers were removed with Trimmomatic v. 0.39 (Bolger et al., 2014) and BBduk tools (Bushnell et al., 2017). Taxonomic assignment of operational taxonomic units (OTUs) was carried out with EDGE bioinformatics web based analysis (Li et al., 2017). We filtered all OTUs with a minimum of 10 sequence reads. We prepared a separate data set and included all reads related to the potential notable animal or human diseases OTUs. We removed mitochondrial 16S rRNA sequences using the prune_taxa function. Alpha diversity was calculated using the Chao 1 index and Shannon measure with absolute abundance of reads in phyloseq v. 1.46.0 (McMurdie and Holmes, 2013). Differences between the microbiota of cloacal and tracheal samples of each individual were estimated using the Anosim function and Mann-Whitney test, and correlations between nesting sites and microbiome composition were measured with Mantel statistics VEGAN v. 2.4-6 (Oksanen et al., 2013). Venn diagrams were drawn using the web tool.^2^

We measured Spearman’s rank-order correlation for detection strength and direction of association between ranked variables: abundance of OTUs in cloacal and tracheal samples of all individuals with cocor v. 1.1-3 (Diedenhofen and Musch, 2015). The presence of potential zoonotic pathogens was estimated according to the CDC pathogen list (Benskin et al., 2009; Vogt, 2023).

## Results

Among the seven birds, four were females and three were males. Approximately 3,500 OTUs were identified from each sample supported by ∼2.8 Million (M) sequence reads. Filtering OTUs with a minimum of 50 reads per taxon resulted in a subset of 1,075 OTUs for bacterial and archaeal domains for further analysis procedures. This selection ensured us to have a robust dataset, focusing on OTUs with sufficient read coverage for reliable analysis. Overall, 1,075 species belonged to 193 families, 51 classes, and 24 phyla. Most taxa were classified as Pseudomonadota (42.6%), Bacillota (22%), and Actinemicetota (14.49%) phyla. No significant differences in bacterial composition were observed between sexes.

**FIGURE 1:**
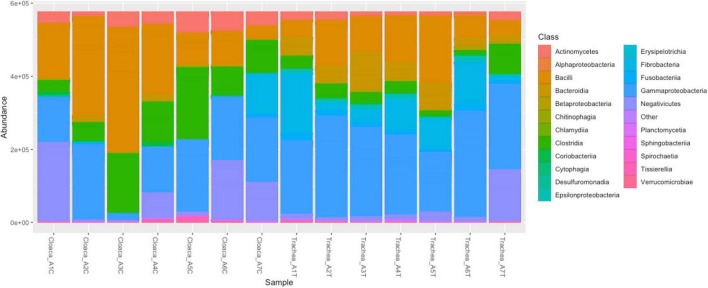
Composition of bacterial classes in cloacal and tracheal samples of *Aquila heliacal.* Abundances are measured using bray-curtis distances. Each color corresponds to a bacterial class, and the vertical bars represent the abundance of species at the class level. The X-axis denotes the samples.

The relative abundance of taxonomic classes in the bacterial community is shown in Figure 1. The most abundant bacterial classes in the tracheal and cloacal samples of *A. heliaca* were *Gammaproteobacteria* (67.5%), *Bacilli* (43.8%), and *Negativicutes* (22.0%). The following classes were detected in higher proportions in the cloaca: *Negativicutes* (16.69%), *Clostridia* (16.1%), and *Actinomycetes* (6.21%), while *Fusobacteria* (15.4%), *Bacteroidia* (9.6%), and *Betaproteobacteria* (9.6%) were detected in the tracheal samples.

**FIGURE 2:**
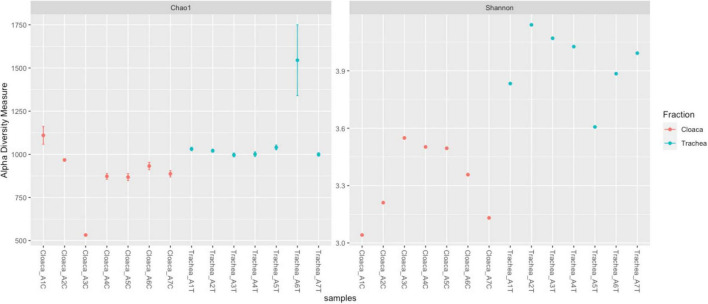
Alpha diversity plot visualizing differences in microbiota structure across samples. The Y-axis represents alpha diversity measures, while the X-axis distinguishes between samples (red for cloacal, blue for tracheal). Diversity is assessed using Chao1 and Shannon indexes.

**FIGURE 3:**
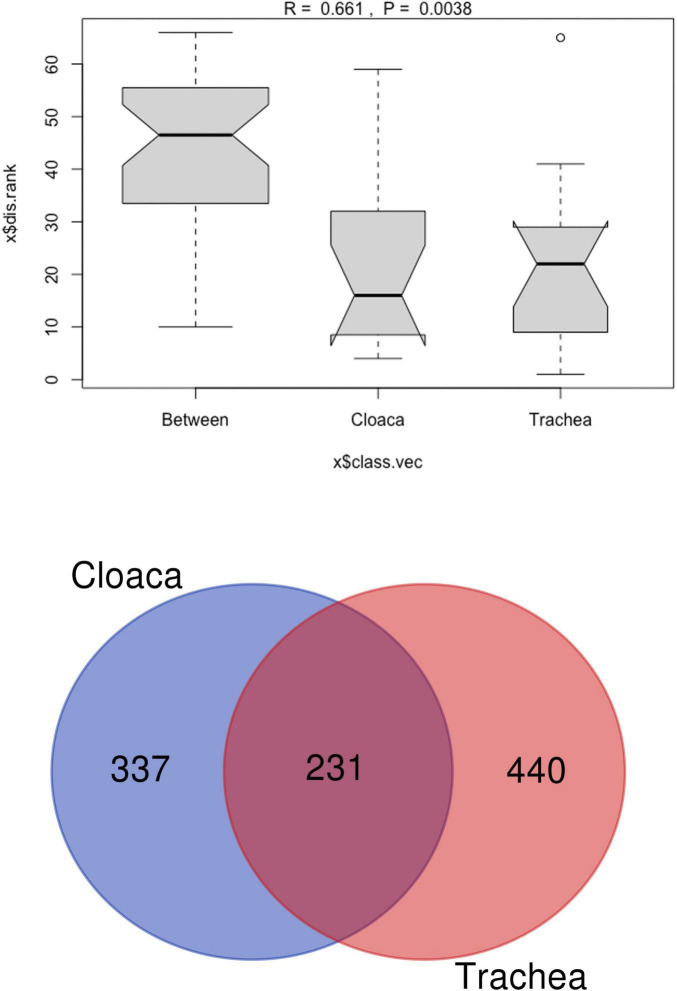
**(a)** Analysis of similarity (ANOSIM) plot testing dissimilarity between cloacal and tracheal bacterial communities in *A. heliaca* samples. The Y-axis represents the distance rank between samples, and the X-axis represents the dissimilarity results between both groups. **(b)** Venn diagram for cloacal and tracheal bacterial species in Eastern Imperial Eagle samples. The Venn diagram shows the total and overlapping (purple) species in the cloaca (blue) and trachea (pink).

The richness of the bacterial community is reflected by Chao 1 index, which ranges between 500 and 1,075 in different samples. The Shannon index, showing the bacterial community diversity, ranged from 3.0 to 4.0 (Figure 2). Comparison of tracheal and cloacal bacterial communities revealed a significant difference according to the Kruskal-Wallis test (49.56, *p* = 0); Mann-Whitney *U*-Test (*p* = 0.001). The tested individuals shed distinctive microbial communities in their digestive and respiratory tracts, and dissimilarity was measured with Anosim R 0.45; *P* < 0.005 (Figure 3a). The length of the bows represents the level of heterogeneity, and the width represents the number of screening species. Among the identified taxa 440 OTUs were unique for the trachea and 337 OTUs were unique for the cloaca, while 21.5% species were shared by cloacal and tracheal communities, Venn diagram (Figure 3b). Analysis of cumulative taxon abundances in the trachea and cloaca indicated significant evidence of moderate positive monotonic relationship between cloacal and tracheal bacterial communities (Spearman’s rank correlation coefficient rho = 0.67, *p* < 2.2e −16).

No statistically significant differences in microflora composition were detected between individuals from different nests when tracheal and cloacal samples were compared separately (Anosim tracheal samples *R* = 0.39, *p* = 0.2; Anosim cloacal samples *R* = 0.14, *p* = 0.5). However, the bacterial composition in tracheal samples showed a significant correlation with geographic distance (Mantel statistic *r* = 0.75, *p* = 0.03), whereas no significant correlation was found in cloacal samples (Mantel statistic *r* = −0.04, *p* = 0.4).

Among the identified bacterial species, 382 have the potential to cause notable human and wildlife diseases. The most abundant bacterial species was *Alteromonas australica*, represented by 1 million reads. The tracheal and cloacal microbiomes were dominated by *Staphylococcus aureus, Megamonas funiformis*, and *Ornithobacterium rhinotracheale.* The following pathogenic species were detected in the cloaca only: *Staphylococcus simulans, Acinetobacter radioresistens, Fastidiosipila sanguinis, Trueperella bialowiezensis*, and *Mycoplasma iowae*. Pathogens such as *Moraxella cuniculi, Moraxella ovis, Pasteurella aerogenes, Bacteroides vulgatus, Campylobacter gracilis, Mannheimia varigena, Massilia oculi, Mycoplasma phocidae, Fusobacterium ulcerans, Weeksella virosa, Mycoplasma maculosum, Morganella, Morganii, Helicobacter pylori, Neisseria canis, Xanthomonas vasicola*, and *Yersinia enterocolitica* originate from the trachea and was not detected in cloacal samples.

In our dataset, we identified four out of five species from the HACEK group, (*Haemophilus parainfluenzae*, *Aggregatibacter aphrophilus*, *Cardiobacterium hominis*, and *Eikenella corrodens*) of gram-negative bacteria associated with infective endocarditis in humans (Bläckberg et al., 2021; Khaledi et al., 2022). We identified widely known zoonotic bacterial species, such as *Salmonella* spp., *Klebsielle* spp., *Campilobacter* spp., and *Chlamydia psittaci.* Host-specific mammalian pathogens include *Bacillus anthracis*, *Mycobacterium leprae*, *Brucella abortus*, *B. canis*, *B. melitensis*, *Streptococcus pyogenes*, *Vibrio cholerae, V. mimicus*, and Halophilic *Vibrio cincinnatiensis*.

## Discussion

We present the first insights into the microbiome inventory of Eastern Imperial Eagles. The species is VU worldwide (IUCN, 2016). We characterized the tracheal and cloacal microbiomes of 30% of the Georgian population using the complete 16S rRNA gene. Taxonomic assignment of bacterial taxa using the 16S rRNA sequencing method is a cornerstone of taxonomic identification, as highlighted by recent studies (Jeong et al., 2021; Abellan-Schneyder et al., 2021; Hung et al., 2022). Although the number of wildlife microbiome studies is growing, the majority concentrate on mammals (Colston and Jackson, 2016; Moeller and Sanders, 2020), commercially significant birds, and captive-bred avian species (Zhao et al., 2022). Studies investigating the microbiomes of free-living raptors face limitations such as small population sizes, uncontrolled environmental factors (including diet, microhabitats, age, and season), and the difficulties associated with capturing and sampling (Oliveira et al., 2020).

In our study, we identified approximately 3,500 OTUs per sample from ∼2.8 million sequence reads. After filtering for OTUs with a minimum of 50 reads per taxon, we retained 1,075 OTUs, representing bacterial and archaeal domains. These OTUs were classified into 193 families, 51 classes, and 24 phyla. Microbial composition and diversity of raptors is still poorly understood. However, studies on other bird species offer some context. For instance, Kursa et al. (2021) identified 144 bacterial genera in the upper respiratory tract of 507 turkeys. Costanzo et al. (2022) screened 1,700-32,000 operational taxonomic units (OTUs) from 19 phyla in the cloaca of cavity-nesting lesser kestrels (*Falco naumanni*). Similarly, Gao et al. (2021) found 26,000 OTUs, classified into 32 phyla, in the fecal microbiota of six waterbird species. When applied to threatened species, microbiome studies become particularly potent, offering a window into the microbiome that can shed light on critical aspects of host health and survival as the microbiome plays a pivotal role in shaping host fitness. Currently, threats to biodiversity such as climate change, habitat degradation, antimicrobial resistance, and the emergence of infectious diseases, make understanding and managing the microbiome of species of heightened importance (Bahrndorff et al., 2016; West et al., 2019; Akoijam and Joshi, 2022). These risks often induce shifts in microbial communities associated with hosts across all taxonomic groups (Trevelline et al., 2019).

According to our dataset, both the tracheal and cloacal communities showed diversity of bacterial species. The richness of the bacterial community (alpha diversity reflected by the Chao 1 index) suggests variability and complexity of bacterial taxa within the studied environments, which is largely expected given their different physiological functions: the trachea hosts bacterias adapted to respiratory functions, while the cloacal bacterial community is associated with digestive and reproductive processes. Migratory birds harbor a wider variety of microorganisms than resident species, due to adaptations related to long-distance travel between breeding and wintering sites (Zhang et al., 2021; Grond et al., 2023).

Mann-Whitney U test and Kruskal-Wallis test showed significant differences in abundance of bacterial species between tracheal and cloacal body sites. This outcome was expected due to the differences in the studied environment: tracheas oxygen-rich conditions contrary to anaerobic cloaca; trachea’s relatively neutral pH vs. cloaca’s alkaline pH; distinct enzymatic profiles, etc. Besides these environmental differences, 21.5% of the identified bacterial species were shared by tracheal and cloacal samples and were dominated by species from *Gammaproteobacteria*, *Bacilli, Clostridia*, and *Negativicutes* taxa. These four bacterial classes were the most prevalent assigned amplicon sequences and overall, 77.0% of the total 16S rRNA reads were detected across our samples. Although these taxa were abundant in both body parts, their relative proportions varied among the samples. This variability can be influenced by genetic factors, temporal dynamics, and potential sampling bias.

Comparison of tracheal samples apart from cloacal samples by Mantel statistics showed that eaglets living in short geographical distances harbored more similar microbiomes than those who were nested further apart. Interestingly, this trend was not observed in cloacal samples. Besides that nests were located alongside the same Iori river in semi-arid habitat and distance between nests was 3.7-96.2 km. The composition of tracheal bacterial species was shaped by microclimate (two nests were located next to the dumpster and four in the river plains) and at this stage of life the birds might be less dependent on the individual parameters of the host. The relatively stable environment (temperature, pH, oxygen level, etc.) in the cloaca might alter similarities in cloacal bacterial composition by stabilizing selection pressure. These factors create a unique microenvironment that can favor the growth and survival of certain bacterial species over others. Through ongoing interactions between host physiology, microbial communities, and environmental conditions, specific bacterial taxa may thrive, while others are suppressed or eliminated. This process of active selection pressure within the cloaca could lead to similar patterns of bacterial composition among individuals of the same populations. Moreover, studies have highlighted the convergence of microbiota in birds with the same diet preferences (Grond et al., 2018; Oliveira et al., 2020; Bodawatta et al., 2022a). We expected qualitative similarities among the studied individuals, as this trend was also detected by other studies (Crespo et al., 2019; de Jonge et al., 2022), and slight variation in the bacterial species composition in the cloaca of *A. heliaca* chicks could be explained by interindividual differences.

*Negativicutes* (16.7%), *Clostridia* (16.1%), and *Actinomycetes* (6.2%) were prevalent in the cloaca, while *Bacteroidia* (9.6%), *Fusobacteria* (15.4%), and *Betaproteobacteria* (9.5%) were enriched in the trachea. Notably, the trachea harbored a slightly richer bacterial community, with 440 OTUs, compared to 337 OTUs in the cloaca.

Spearman’s rank correlation coefficient revealed a positive association between the abundance of OTUs in the trachea and cloaca. This suggests that tracheal microbiomes reflects cloacal microbiomes, particularly for abundant taxa such as *Gammaproteobacteria*, *Bacilli*, *Clostridia*, and *Negativicutes*. Hence, sampling the trachea and cloaca could be a reasonable approach to estimate the overall bacterial composition. However, it is important to recognize that sampling either the trachea or cloaca may not provide a comprehensive understanding of the microbiota, particularly with regard to pathogenic bacterial species and those that could pose zoonotic risks. Furthermore, the correlation with less abundant taxa may be weaker.

The observed similarities in overall bacterial composition (trachea plus cloaca) is more likely due to close geographic locations, similar dietary habits, microclimate of nests than small sample size. Although we recognize the potential limitations of our study, larger, more complex investigations with differing age groups, varied habitats are needed to deepen our understanding and potentially revise our current conclusions. Further research is crucial to fully determine the drivers of *A. heliaca* microbiome composition.

### Bacterial pathogens

Based on our results, commensal and pathogenic bacterial species belong predominantly to the phyla Pseudomonadota, Bacillota, and Actinemicetota. These taxa have also been reported in other bird species (Benskin et al., 2009; Colston and Jackson, 2016; Bahrndorff et al., 2016; Grond et al., 2018; Capunitan et al., 2020). Eastern imperial eagles primarily prey on rodents, which often carry pathogenic bacteria. Additionally, during long breeding periods, the accumulation of feathers, skin remains and pellets in the nest creates a conducive environment for bacterial growth. These factors explain the existence of 382 pathogenic bacterial species in our samples. Blautia, Enterococci, Staphylococci, and Streptococci are common bacterial taxa in the respiratory tract (Bosch et al., 2013; Shabbir et al., 2015; Faldynova et al., 2024) and are widespread among our sampled birds.

*Alteromonas australica* (represented by the highest number of reads) is associated with seawater and marine sediments (López-Pérez et al., 2014). Unexpectedly, this bacterial species was not detected as part of the tracheal or cloacal microbiome; however, the abundance of the reads could be attributed to the capacity of Alteromonas to rapidly proliferate in the presence of suitable substrates (Chen et al., 2020). The opportunistic pathogens *Staphylococcus aureus, Megamonas funiformis*, *M. hypermegale*, and *Ornithobacterium rhinotracheale* had the most reads in the studied tracheal and cloacal samples. *Staphylococcus* is a normal flora on the mucous membrane and skin but also has a clumping factor and forms blood clots (Yehia et al., 2023). *Staphylococcus aureus* can cause skin and respiratory tract infections in birds, especially those under stress or with compromised immune systems (Ferrer and Hiraldo, 1995; Silva et al., 2022). Both Megamonas species are found in the digestive tract and are considered part of the gut microbiome (Bodawatta et al., 2022a,b).

*Ornithobacterium rhinotracheale, Mycoplasma gallisepticum, Pasteurella multocida, Riemerella anatipestifer*, and *Bordetella avium* cause respiratory infections with high mortality rates among poultry, particularly during the first month (Barbosa et al., 2019). In addition to these avian respiratory pathogens, we detected the following respiratory disease agents in tracheal samples: five Mycoplasma species (*M. californicum*, *M. gallinaceum*, *M. iowae*, *M. maculosum*, and *M. phocidae*), three Bordetella species (*B. bronchiseptica, B. parapertussis, and B. pertussis*) and two Pasteurella species (*P. aerogans* and *P. dagmatis*). The transmission of avian mycoplasma through horizontal (between individuals) and vertical (adults to its progeny) routes has been described (Yehia et al., 2023). However, the prevalence, clinical significance, and transmission dynamics of bacteria in eagles are not well understood. Further research is needed to investigate the frequency of occurrence, their impact on health and welfare, and the mechanisms by which they are transmitted among *A. heliaca* individuals.

The following opportunistic pathogenic species were detected in the cloaca only: *Staphylococcus simulans, Acinetobacter radioresistens, Fastidiosipila sanguinis*, and *Trueperella bialowiezensis*. *Moraxella cuniculi, M. ovis, Bacteroides vulgatus, Campylobacter gracilis, Mannheimia varigena, Massilia oculi, Fusobacterium ulcerans, Weeksella virosa, Morganella, Morganorganii, Helicobacter pylori, Neisseria canis, Xanthomonas vasicola*, and *Yersinia enterocolitica* originated from the trachea and could not be detected in cloacal samples.

Various *Salmonella* species have been detected in both domestic and wild birds (Belo et al., 2023; Smith et al., 2023). We detected *S. bongori* in all the samples and *Salmonella enterica* in four eaglets. *S. bongori* is known in reptiles, and few cases of human infection have been described (Fookes et al., 2011). On the other hand, *Salmonella enterica* includes a wide range of servers and is responsible for various types of human and animal illnesses and causes of food-borne illness worldwide (Belo et al., 2023; Smith et al., 2023). Understanding the prevalence and diversity of Salmonella species in threatened eagles is important for effective conservation, disease management and public health efforts.

We detected three species: *Klebsiella aerogens, K. pneumonia*, and *K. variicola* in our dataset. Among them, pathogenic *K. variicola* represented approximately 5K reads in each sample. The high level of abundance suggests its potential significance in the sampled population. *K. variicola* infects various hosts, and people infected with a high titer of *K. variicola* have a high mortality rate (Maatallah et al., 2014). *K. pneumoniae* is a component of the normal intestinal flora but has the potential to infect the respiratory tract in humans. *K. aerogenes* is associated with respiratory and urinary tract infections (Rodríguez-Medina et al., 2019). The presence of these Klebsiella species, particularly pathogenic *K. variicola*, raises concerns about potential health risks to both humans and animals.

In the present study, some species of bacilli were found to be highly prevalent in *B. mycoides* in two cloacal and one tracheal sample and in *B. coagulans* in one tracheal sample. Additionally, *B. cereus*, *B. subtilis*, and *B. thuringiensis* were also identified. However, to the best of our knowledge, *B. anthracis* has not been previously attributed to the respiratory or digestive system of eagles.

Campylobacteriosis is the most commonly reported zoonotic bacterial food-borne gastroenteritis worldwide (Igwaran and Okoh, 2019). Eleven species were identified in this study (*Campylobacter coli, C. concisus, C. curvus, C. fetus, C. gracilis, C. helveticus, C. jejuni, C. lari, C. showae, C. upsaliensis*, and *C. ureolyticus*). Among them, *C. concisus* was present in all tracheal samples analyzed. Different species of Campylobacter have been isolated from wild and domestic birds worldwide, due to their pathogenicity and antibiotic resistance, this is currently a global challenge (Ahmed and Gulhan, 2022).

Ten *Leptospira* species with low abundance were detected in our dataset: *L. alexanderi*, *L. borgpetersenii L. fainei*, *L. inadai L. broomii, L. kirschneri, L. meyeri, L. noguchii, L. santarosai, and L. weilii*. Most of the identified species are associated with leptospirosis. Epidemiological studies on leptospirosis in wild birds and its role in transmission to other animals are rare (Balcázar et al., 2024).

*Yersinia enterocolitica* was detected in only one tracheal sample, while *Y. pseudotuberculosis* and *Y. freundiksenii* were detected in all the samples. *Vibrio mimicus* was identified from only one cloacal sample, but *V. cholerae*, *V*. *cincinnatiensis, V. mimicus*, and *V. parahaemolyticus V. vulnificus* were identified across all the samples. The detection of *Yersinia* and Vibrio spps. suggests that *A. heliaca* might be a carrier, potential reservoir, or source of infection.

To determine whether the abundance of pathogens in the cloaca and trachea is sufficient to cause infections, several factors must be considered, including the type of pathogen, its concentration, and the overall health of the host. The mere presence of certain pathogens in these sites does not automatically indicate active disease. While the existence of a pathogenic agent could potentially lead to infection, it may not always cause development of disease unless factors such as stress, poor nutrition, a compromised immune system, or other environmental conditions are present. Therefore, this balance should be carefully considered, taking into account both the potential risks of infection and the welfare of the host when designing study protocols.

## Conclusion

*Aquila heliaca* can be considered as a reliable source of the presence of pathogenic bacterial species in their respective habitats. However, capturing them for microbiome studies poses a significant challenge, due to elusive nature, low abundance, difficulties in safely handling and vulnerability. Therefore, during conservation activities such as ringing or tagging with telemetry devices, it is imperative to prioritize sample collection for microbiome studies. Efforts aiming at conservation of red-listed birds may not presently focus on microbiome considerations but integrating microbiome research into conservation strategies could yield significant benefits. Understanding bacterial abundance through such studies provides valuable insights into the health of the species, as disease outbreaks have the potential to disrupt ecological interactions, impact reproductive capabilities, alter predator-prey dynamics, decrease population sizes, and even lead to species extinction. Consequently, monitoring efforts are essential for assessing the risk of zoonotic disease transmission to humans and implementing appropriate control measures to prevent the spread of infection among other species. Therefore, integrating microbiome monitoring into ongoing conservation efforts is not only beneficial for the species under study but also for understanding broader ecological health. Ultimately, it provides a comprehensive approach to species conservation that includes the often-overlooked role of microbial ecosystems in the overall wellbeing of ecosystems.

## Data Availability

The dataset analyzed in this study is available in the NCBI Sequence Read Archive (SRA) under BioProject accession number PRJNA1123231.

## Footnotes

1
https://www.geneious.com

2
http://bioinformatics.psb.ugent.be/webtools/Venn/
